# Audit on the Assessment of Patients by the Primary Care Mental Health Practitioner at the Brentwood Community Centre and Subsequent Referral Pathways from January 2023 till January 2024

**DOI:** 10.1192/bjo.2025.10647

**Published:** 2025-06-20

**Authors:** Ihuoma Queen Oka, Ajay Kurien, Vishal Agrawal

**Affiliations:** 1 School – East of England. Essex Partnership University Foundation NHS Trust, Essex, United Kingdom; 2 Essex Partnership University Foundation NHS Trust, Essex, United Kingdom

## Abstract

**Aims:** The general aim of the audit is to identify the assessment procedure and subsequent referrals of patients making contact with the Primary Care Practitioner at the Brentwood Community Centre.

**Methods:** All the patients who were assessed by the Primary Care Practitioner attached to The Brentwood Resource Centre from January 2023 till January 2024 in order of attendance were selected consecutively.

Audit standards:

1. All new referrals should be first seen by the Mental Health Practitioner.

2. MHP assessment template – MHP – PCN Consultation V2

Sampling: A list of all the patients who were assessed by the Primary Care Practitioner attached to The Brentwood Resource Centre in order of attendance were selected consecutively for the time period. Sample size was 776.

Data collection: Data was collected retrospectively from the operating system (Mobius) using a data collection tool.

Setting: Community Mental Health Team, Brentwood.

Inclusion criteria:

1. Adult patients aged 18–70.

2. All adult patients as stated above that presented to the Primary Mental Health Care Practitioner at the Brentwood Resource Centre.

3. Patients who were within the catchment area of the Primary Care Network for The Brentwood Resource Centre.

Exclusion criteria:

1. Patients aged below 18 years.

2. Patients aged above 70 years.

3. Patients who did not fall within the catchment area of the Primary Care Network for the Brentwood Resource Centre.

Data handling and analysis: SPSS Version 27 was used for data entry and analysis.

Time duration: The data collection and analysis was be completed in 3 months of obtaining approval.

**Results:** The target aim was for a 100% compliance however the compliance was less than 100%.

Overall referrals that were initially assessed – 69.2% (82.5%).

Referrals from GP that were compliant with audit standards – 67.2% (80.2%).

Assessment using MHP Assessment template – 100%.

These were because some of the patients referred did not engage with the service 190 (24.5%) and 44 (5.7%) of data were not available to be analysed.

**Conclusion:** Details of good practice:

1. All referrals made were screened and timely invites sent to patients for further assessments.

2. Outcomes of assessments were clearly documented.

3. Trust protocols for referrals i.e. discussions in MDT were followed.

Areas of improvement:

1. Clear documentations of outcomes for referrals made to the service and patients did not engage.

2. The use of the MHP Assessment template – MHP PCN Consultation V2 and uploading to the correct platform on operating software of the service.

